# Biliary Calculi and Olive Oil

**Published:** 1890-01-11

**Authors:** 


### BILIARY CALCULI AND OLIVE OIL.

About fifteen years ago a homœopathic practitioner in America first introduced the treatment of biliary calculi by means of large doses of olive oil, which, with or without belladonna, is almost exclusively employed in that country, and has been so to a less extent in Europe. It is certain that it does excite an enormous secretion of bile which, distending the gall duct, overcomes the obstruction to which the agonising pain is due, and, as might be expected, the gall-stones are frequently found in the stools; but notwithstanding the cautions of Ball, Flint, and Shingleton Smith, many medical men have fallen into the error of mistaking the masses of inspissated oil, which are abundantly passed, for softened calculi. But, without going so far, there is no question as to the startling success that follows this treatment in many cases, "tumours" disappear and colic and jaundice cease, if not permanently, at any rate for months or years. The only difficulty is the repugnance which most patients feel to swallowing enormous doses of oil, and the refusal of the stomach to retain them. Dr. Siegfried Rosenburg has recently given a summary of the observations of others and of his own experience. He has employed it with the best results in twenty-one cases, and has overcome the difficulties connected with its administration by disguising its flavour, colour, and characters. He generally gives it in doses of 100-200 grams. (between quarter and half pint) repeated, if necessary, after two to four days, adding to the oil 25 per cent. of menthol, 10-15 per cent. of cognac, and the yelks of two eggs. The menthol, besides its strong taste, tends by its anæsthetic action on the stomach to avert the vomiting, and the absence of desire or relish for food, which does not last for more than twenty-four hours, is the only unpleasant effect complained of. He rarely found any benefit from the much-vaunted Carlsbad waters, but from experiments on animals, as well as observations on man, he feels justified in recommending salicylate of soda in large doses of hot water as a really efficient cholagogue.

